# Supplementary material for: Differences in gene expression in field populations of Wolbachia-infected Aedes aegypti mosquitoes with varying release histories in northern Australia
Source: PLoS Negl Trop Dis. 2023 Mar 29;17(3):e0011222. doi: 10.1371/journal.pntd.0011222 (PMC10085034; doi:10.1371/journal.pntd.0011222)

Supporting information

S1 Fig. Density of *Wolbachia* in *Aedes aegypti* descended from mosquitoes released in the Cairns region of northern Australia in 2013-14 and 2017. Each dot is an individual mosquito and bars and whiskers are medians and 95% confidence intervals, respectively. There was no significant difference ($P > 0.05$; Mann-Whitney U test) in *Wolbachia* density between the years.

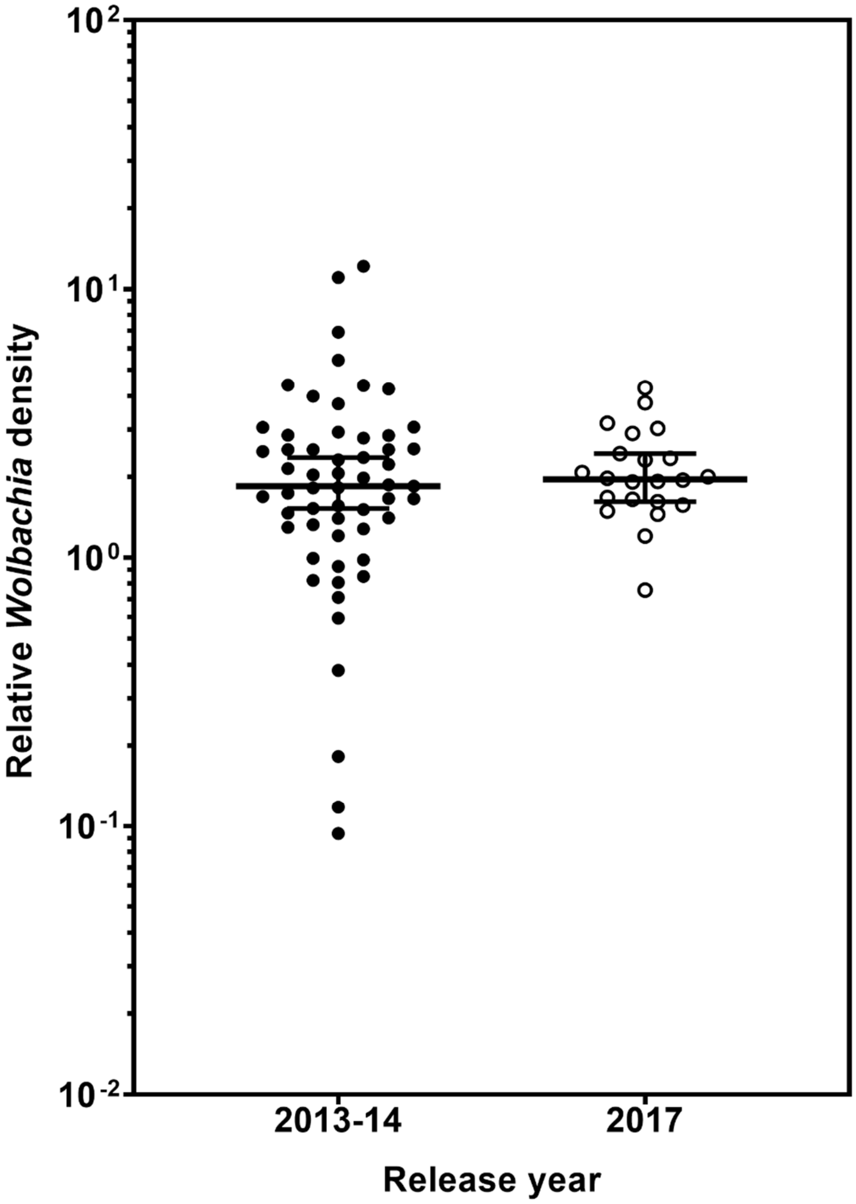

Supplement: S1 Fig — Each dot is an individual mosquito, and bars and whiskers are medians and 95% confidence intervals, respectively. There was no significant difference (P > 0.05; Mann-Whitney U test) in Wolbachia density between the years. (PDF) [file pntd.0011222.s001.pdf]
