# Supplementary material for: Differences in gene expression in field populations of Wolbachia-infected Aedes aegypti mosquitoes with varying release histories in northern Australia
Source: PLoS Negl Trop Dis. 2023 Mar 29;17(3):e0011222. doi: 10.1371/journal.pntd.0011222 (PMC10085034; doi:10.1371/journal.pntd.0011222)
Supplement: S1 Table — (PDF) [file pntd.0011222.s002.pdf]

S1 Table. **Mapping statistics.**

Sample ID	Raw reads _Lane 1	Raw reads _Lane 2	Read pairs after quality and adapter trimming	Number of aligned read pairs	% of mapped reads
**210290**	34,582,326	34,261,303	48,817,765	43,369,702	88.84
**210291**	32,231,667	31,968,205	49,766,245	43,923,688	88.26
**210295**	33,580,572	33,244,380	46,137,538	41,094,705	89.07
**210297**	31,131,370	30,809,360	44,690,792	39,497,722	88.38
**210298**	30,903,897	30,581,705	48,448,379	43,206,264	89.18
**210299**	27,455,578	27,181,427	44,280,347	39,449,361	89.09
**210300**	29,400,828	29,080,319	44,257,287	39,327,025	88.86
**210301**	29,959,142	29,631,518	43,956,040	39,107,689	88.97
**210304**	32,320,363	31,988,121	42,862,574	38,387,721	89.56
**210306**	29,532,465	29,238,980	45,484,308	40,617,487	89.3
**210307**	27,631,383	27,344,243	43,284,712	38,791,759	89.62
**210308**	29,086,571	28,792,458	46,165,028	41,451,579	89.79
**210309**	31,162,958	30,886,606	49,677,557	44,660,124	89.9
**210310**	33,538,179	33,263,415	50,971,064	45,583,423	89.43
**210311**	30,444,038	30,183,667	47,816,102	42,728,469	89.36
**210312**	33,082,679	32,814,398	52,004,903	46,606,794	89.62
**210313**	27,652,952	27,402,701	43,698,630	38,935,479	89.10
**210315**	30,844,599	30,545,487	46,584,306	41,571,835	89.24
**210318**	34,698,571	34,346,127	50,984,466	45,600,506	89.44
**210319**	28,562,993	28,300,042	45,036,623	40,073,587	88.98
**210320**	33,586,176	33,226,843	45,484,408	40,858,644	89.83
**210323**	29,810,832	29,520,295	52,284,923	47,563,594	90.97
**210325**	34,343,425	34,074,248	46,562,372	41,831,635	89.84
**210326**	29,465,230	29,212,789	48,737,908	44,195,535	90.68
**210327**	31,823,328	31,582,004	49,253,762	44,333,311	90.01
**210329**	28,965,050	28,639,100	42,481,903	38,068,033	89.61
**210330**	30,294,268	29,888,141	46,080,368	41,043,784	89.07
**210331**	28,803,611	28,597,283	45,709,413	40,311,131	88.19
**210332**	30,430,171	30,053,350	48,847,663	43,874,971	89.82
**210333**	35,172,080	34,778,341	58,624,619	52,685,945	89.87
**210335**	32,188,560	31,805,573	43,615,162	39,044,293	89.52
**210336**	33,499,898	33,145,782	48,487,016	43,536,492	89.79
**210338**	29,651,131	29,191,005	43,757,822	38,979,468	89.08
**Total**	1,025,836,891	1,015,579,216	1,554,852,005	1,390,311,756	89.42
**Final total**	2,041,416,107				
