# Supplementary material for: Differences in gene expression in field populations of Wolbachia-infected Aedes aegypti mosquitoes with varying release histories in northern Australia
Source: PLoS Negl Trop Dis. 2023 Mar 29;17(3):e0011222. doi: 10.1371/journal.pntd.0011222 (PMC10085034; doi:10.1371/journal.pntd.0011222)
Supplement: S2 Table — (PDF) [file pntd.0011222.s003.pdf]

S2 Table. Pairwise comparison of median gene expression (CPM) according to release history.

**Pairwise comparison of CPM**	**Mean difference**	***P*-value***
$Aae.wMel_{2017}$ vs $Aae.wMel_{2013/2014}$	9035.44 vs 8161.84	<0.001
$Aae.wMel_{2013/2014}$ vs $Aae.wMel_{2011}$	8161.84 vs 8158.72	0.970
$Aae.wMel_{2017}$ vs $Aae.wMel_{2011}$	9035.44 vs 8158.72	<0.001

P* value based on post-hoc analysis of Kruskal-Wallis test.
