# Supplementary material for: Differences in gene expression in field populations of Wolbachia-infected Aedes aegypti mosquitoes with varying release histories in northern Australia
Source: PLoS Negl Trop Dis. 2023 Mar 29;17(3):e0011222. doi: 10.1371/journal.pntd.0011222 (PMC10085034; doi:10.1371/journal.pntd.0011222)
Supplement: S3 Table — (PDF) [file pntd.0011222.s004.pdf]

S3 Table. KEGG pathway enrichment analysis of 357 commonly upregulated DEGs across all release years.

A. Using DAVID Bioinformatics tool

#Term ID	Term description	Observed gene count	Background gene count	False discovery rate	Matching proteins in your network (IDs)	Matching proteins in your network (labels)
aag00260	Glycine, serine and threonine metabolism	6	27	8.61E-05	7159.AAEL000213-PA, 7159.AAEL005336-PA, 7159.AAEL010276-PA, 7159.AAEL012578-PA, 7159.AAEL012764-PA, 7159.AAEL014426-PA	AAEL000213, AAEL005336, AAEL010276, AAEL012578, AAEL012764, AAEL014426
aag01230	Biosynthesis of amino acids	7	48	8.61E-05	7159.AAEL002675-PA, 7159.AAEL003345-PA, 7159.AAEL004701-PA, 7159.AAEL005336-PA, 7159.AAEL006834-PA, 7159.AAEL008963-PA, 7159.AAEL012578-PA	AAEL002675, AAEL003345, AAEL004701, AAEL005336, AAEL006834, AAEL008963, AAEL012578
aag01100	Metabolic pathways	22	716	0.00088	7159.AAEL000024-PA, 7159.AAEL000213-PA, 7159.AAEL000642-PA, 7159.AAEL001194-PA, 7159.AAEL001757-PA, 7159.AAEL002194-PA, 7159.AAEL002269-PA, 7159.AAEL002675-PA, 7159.AAEL002683-PA, 7159.AAEL003345-PA, 7159.AAEL004701-PA, 7159.AAEL005336-PA, 7159.AAEL005769-PA, 7159.AAEL005790-PA, 7159.AAEL006834-PA, 7159.AAEL008963-PA,	AAEL000024, AAEL000213, AAEL000642, AAEL001194, AAEL001757, UO, AAEL002269, AAEL002675, AAEL002683, AAEL003345, AAEL004701, AAEL005336, AAEL005769, AAEL005790, AAEL006834, AAEL008963, AAEL009679, AAEL010276, AAEL012578, AAEL013637, AAEL014426, AAEL014556

					7159.AAEL009679-PA, 7159.AAEL010276-PA, 7159.AAEL012578-PA, 7159.AAEL013637-PA, 7159.AAEL014426-PA, 7159.AAEL015053-PB	
aag00220	Arginine biosynthesis	3	13	0.008	7159.AAEL002675-PA, 7159.AAEL003345-PA, 7159.AAEL004701-PA	AAEL002675, AAEL003345, AAEL004701
aag00232	Caffeine metabolism	2	3	0.0107	7159.AAEL002194-PA, 7159.AAEL002683-PA	UO, AAEL002683
aag00350	Tyrosine metabolism	3	17	0.0107	7159.AAEL000024-PA, 7159.AAEL008963-PA, 7159.AAEL013637-PA	AAEL000024, AAEL008963, AAEL013637
aag01200	Carbon metabolism	5	78	0.0172	7159.AAEL005336-PA, 7159.AAEL005790-PA, 7159.AAEL010276-PA, 7159.AAEL012578-PA, 7159.AAEL014426-PA	AAEL005336, AAEL005790, AAEL010276, AAEL012578, AAEL014426
aag00330	Arginine and proline metabolism	3	26	0.0234	7159.AAEL000213-PA, 7159.AAEL002675-PA, 7159.AAEL006834-PA	AAEL000213, AAEL002675, AAEL006834
aag00330	Arginine and proline metabolism	3	26	0.0301	7159.AAEL003116-PA, 7159.AAEL005575-PA, 7159.AAEL008108-PA	AAEL003116, AAEL005575, AAEL008108
aag04745	Phototransduction - fly	3	30	8.61E-05	7159.AAEL000213-PA, 7159.AAEL005336-PA, 7159.AAEL010276-PA, 7159.AAEL012578-PA, 7159.AAEL012764-PA, 7159.AAEL014426-PA	AAEL000213, AAEL005336, AAEL010276, AAEL012578, AAEL012764, AAEL014426

B. Using KEGG mapper

KEGG BRITE identifier, description (no. of hits in data set	NCBI Gene ID and description
aag00001 KEGG Orthology (129)	5576561 Isocitrate dehydrogenase [NADP], mitochondrial; 5567033 glucose dehydrogenase [FAD, quinone]; 5568730 FGGY carbohydrate kinase domain-containing protein; 5565449 maltase A3; 5570982 inositol oxygenase; 5577552 acidic endochitinase SP2; 5567037 NADP-dependent malic enzyme isoform X1; 5564415 LOW QUALITY PROTEIN: glycine dehydrogenase (decarboxylating), mitochondrial; 5573084 aminomethyltransferase, mitochondrial; 5568814 fatty acid synthase; 5573929 fatty acid synthase; 5570274 elongation of very long chain fatty acids protein 4; 5575620 elongation of very long chain fatty acids protein 7; 5563869 elongation of very long chain fatty acids protein 7; 5579999 fatty acyl-CoA reductase wat; 5572250 group 3 secretory phospholipase A2 isoform X2; 5574470 esterase B1; 23687459 9 alcohol dehydrogenase 2; 5577524 acyl-CoA Delta(11) desaturase isoform X2; 5574096 purine nucleoside phosphorylase isoform X2; 5575671 xanthine dehydrogenase; 110677659 61 high affinity cGMP-specific 3',5'-cyclic phosphodiesterase 9A-like; 5573896 uricase; 5564672 2-oxo-4-hydroxy-4-carboxy-5-ureidoimidazoline decarboxylase; 5572541 alanine aminotransferase 1; 5565274 argininosuccinate synthase; 5577897 argininosuccinate lyase; 5566353 D-3-phosphoglycerate dehydrogenase; 5576518 probable phosphoserine aminotransferase; 5569995 D-amino-acid oxidase; 5565677 LOW QUALITY PROTEIN: sarcosine dehydrogenase, mitochondrial; 5572760 sarcosine dehydrogenase, mitochondrial; 5576784 glycine N-methyltransferase; 5571325 tyrosine aminotransferase; 5569163 cysteine dioxygenase type 1; 5575552 arginase, hepatic; 5568426 delta-1-pyrroline-5-carboxylate synthase; 5578315 homogentisate 1,2-dioxygenase; 5563570 L-dopachrome tautomerase yellow-f; 110677916 08 selenide, water dikinase 2-like isoform X1; 5575673 glutamyl aminopeptidase; 5572289 iduronate 2-sulfatase; 5577763 membrane-bound alkaline phosphatase; 110673980 89 cytosolic 10-formyltetrahydrofolate dehydrogenase-like; 5568557 probable 4-coumarate--CoA ligase 1; 5567780 luciferin 4-monooxygenase; 5566107 venom carboxylesterase-6; 5574850 cytochrome P450 302a1, mitochondrial; 5575000 perlucin-like protein; 5578720 probable multidrug resistance-associated protein lethal(2)03659; 5579922 vang-like protein 1; 5574685 gonadotropin-releasing hormone II receptor isoform X8; 5576429 low density lipoprotein receptor adapter protein 1-A; 5577378 cathepsin O; 5564141 MD-2-related lipid-recognition protein; 5569420 gram-negative bacteria-binding protein 1; 5571998 peptidoglycan-recognition protein 2; 5577955 uncharacterized protein LOC5577955 isoform X1; 5579094 defensin-C; 5570133 guanine nucleotide-binding protein subunit beta-2; 5566698 transient-receptor-potential-like protein; 5577143 arrestin homolog, 5570893 dual specificity tyrosine-phosphorylation-regulated kinase 4 isoform X1; 5570557 leucine-rich repeat-containing protein 23; 5575574 72 kDa type IV collagenase; 5575549 matrix metalloproteinase-19; 5563550 serine protease easter; 5575056 chymotrypsin-like protease CTRL-1; 5575674 testisin; 5572392 uncharacterized protein LOC5572392; 5579366 polyserase-2; 23687745 0 serine protease easter isoform X1; 5564283 serine protease easter; 5570931 melanization protease 1; 5575054 plasma kallikrein; 5563617 serine protease easter; 110679707 32 serine protease 7-like isoform X1; 5570687 uncharacterized protein LOC5570687; 5568757 transmembrane protease serine 9; 5579360 polyserase-2; 5568004 polyserase-2 isoform X1; 5574108 ovochymase-2; 5563614 transmembrane protease serine 9; 5571775 venom serine carboxypeptidase; 5577347 epoxide hydrolase 4; 5572409 papilin isoform X1; 5572429 CD109 antigen; 23687443 0 CD109 antigen; 5568826 CD109 antigen; 5572428 CD109 antigen; 5564950 acyl-CoA synthetase family member 2, mitochondrial; 5569663 cytochrome P450 4d1 isoform X2; 5571193 probable cytochrome P450 6d5; 5571541 cytochrome P450 6d3; 5570966 LIM/homeobox protein Lhx9; 5566982 forkhead box protein E3

	isoform X3; 5564542 FK506-binding protein 2; 5566491 uncharacterized protein LOC5566491; 110677254 71 homeotic protein spalt-major isoform X1; 23687608 6 protein brown; 5567699 solute carrier family 2, facilitated glucose transporter member 3; 5577476 thiamine transporter 1; 110673989 09 mitochondrial basic amino acids transporter-like; 5570692 LOW QUALITY PROTEIN: mitochondrial basic amino acids transporter; 5572607 uncharacterized protein LOC5572607; 5564315 LOW QUALITY PROTEIN: facilitated trehalose transporter Tret1; 5565788 facilitated trehalose transporter Tret1; 5572577 rootletin isoform X1; 5567446 gelsolin; 5574999 lopap isoform X2; 5578236 apolipoprotein D; 5576882 opsin-3; 5568060 opsin-1; 5572198 opsin, ultraviolet-sensitive; 110680887 55 opsin-1-like; 5567215 pickpocket protein 28; 5565037 bombyxin B-1 homolog isoform X1; 5578083 spondin-1; 5568942 nidogen-2; 5575017 hemicentin-1; 5575607 chorion peroxidase; 5567884 sulfotransferase family cytosolic 1B member 1; 5572213 poly(U)-specific endoribonuclease homolog; 5564209 fatty-acid amide hydrolase 2; 5578864 rutC family protein UK114; 5576269 fatty acid hydroxylase domain-containing protein 2; 5578159 arrestin homolog; 5570483 acidic leucine-rich nuclear phosphoprotein 32 family member A; 110677030 65 leucine-rich repeat-containing protein 40-like
aag01000 Enzymes (81)	5567037 NADP-dependent malic enzyme isoform X1; 5576561 isocitrate dehydrogenase [NADP], mitochondrial; 5566353 D-3-phosphoglycerate dehydrogenase; 23687459 alcohol dehydrogenase 2; 5567033 glucose dehydrogenase [FAD, quinone]; 5568426 delta-1-pyrroline-5-carboxylate synthase; 5579999 fatty acyl-CoA reductase wat; 5569995 D-amino-acid oxidase; 5564415 LOW QUALITY PROTEIN: glycine dehydrogenase (decarboxylating), mitochondrial; 110673980 cytosolic 10-formyltetrahydrofolate dehydrogenase-like, 5565677 LOW QUALITY PROTEIN: sarcosine dehydrogenase, mitochondrial; 5572760 sarcosine dehydrogenase, mitochondrial; 5573896 uricase; 5575607 chorion peroxidase; 5578315 homogentisate 1,2-dioxygenase; 5569163 cysteine dioxygenase type 1; 5570982 inositol oxygenase; 5577524 acyl-CoA Delta(11) desaturase isoform X2; 5574850 cytochrome P450 302a1, mitochondrial; 5571193 probable cytochrome P450 6d5; 5571541 cytochrome P450 6d3; 5569663 cytochrome P450 4d1 isoform X2; 5575671 xanthine dehydrogenase; 5576784 glycine N-methyltransferase; 5573084 aminomethyltransferase, mitochondrial; 5568814 fatty acid synthase; 5573929 fatty acid synthase; 5570274 elongation of very long chain fatty acids protein 4; 5575620 elongation of very long chain fatty acids protein 7; 5563869 elongation of very long chain fatty acids protein 7; 5574096 purine nucleoside phosphorylase isoform X2; 5572541 alanine aminotransferase 1; 5571325 tyrosine aminotransferase; 5576518 probable phosphoserine aminotransferase; 5568730 FGGY carbohydrate kinase domain-containing protein; 110677916 selenide, water dikinase 2-like isoform X1; 5570893 dual specificity tyrosine-phosphorylation- regulated kinase 4 isoform X1; 5567884 sulfotransferase family cytosolic 1B member 1; 5572250 group 3 secretory phospholipase A2 isoform X2; 5574470 esterase B1; 5566107 venom carboxylesterase-6; 5577763 membrane-bound alkaline phosphatase; 110677659 high affinity cGMP-specific 3',5'-cyclic phosphodiesterase 9A-like; 5572289 iduronate 2-sulfatase; 5572213 poly(U)-specific endoribonuclease homolog; 5577552 acidic endochitinase SP2; 5565449 maltase A3; 5577347 epoxide hydrolase 4; 5575673 glutamyl aminopeptidase; 5571775 venom serine carboxypeptidase; 5570931 melanization protease 1; 5575054 plasma kallikrein; 5563617 serine protease easter; 5564283 serine protease easter; 5563550 serine protease easter; 5575056 chymotrypsin-like protease CTRL-1; 5575674 testisin; 5572392 uncharacterized protein LOC5572392; 5579366 polyserase-2; 5570687 uncharacterized protein LOC5570687<br>5568757 transmembrane protease serine 9; 5579360 polyserase-2; 5568004 polyserase-2 isoform X1; 5574108 ovochymase-2; 5563614 transmembrane protease serine 9; 23687745 serine protease easter isoform X1; 110679707 2 serine protease 7-like isoform X1; 5577378 cathepsin O; 5575574 72 kDa type IV collagenase; 5575549 matrix metalloproteinase-19; 5564209 fatty-acid amide hydrolase 2; 5575552 arginase, hepatic

	5578864 rutC family protein UK114; 5564672 2-oxo-4-hydroxy-4-carboxy-5-ureidoimidazoline; 5577897 argininosuccinate lyase; 5564542 FK506-binding protein 2; 5563570 L-dopachrome tautomerase yellow-f; 5564950 acyl-CoA synthetase family member 2, mitochondrial; 5568557 probable 4-coumarate--CoA ligase 1; 5567780 luciferin 4-monooxygenase; 5565274 argininosuccinate synthase
aag01002 Peptidases and inhibitors (28)	5577378 cathepsin O; 5575673 glutamyl aminopeptidase; 5575574 72 kDa type IV collagenase; 5575549 matrix metalloproteinase-19; 5563550 serine protease easter; 5575056 chymotrypsin-like protease CTRL-1; 5575674 testisin; 5572392 uncharacterized protein LOC5572392; 5579366 polyserase-2; 23687745 serine protease easter isoform X1; 5564283 serine protease easter; 5570931 melanization protease 1; 5575054 plasma kallikrein; 5563617 serine protease easter ; 110679707 serine protease 7-like isoform X1; 5570687 uncharacterized protein LOC5570687; 5568757 transmembrane protease serine 9; 5579360 polyserase-2; 5568004 polyserase-2 isoform X1; 5574108 ovochymase-2; 5563614 transmembrane protease serine 9; 5571775 venom serine carboxypeptidase; 5577347 epoxide hydrolase 4; 5572409 papilin isoform X1; 5572429 CD109 antigen;<br>23687443 CD109 antigen; 5568826 CD109 antigen; 5572428 CD109 antigen
aag04147 Exosome (12)	5567446 gelsolin; 5568814 fatty acid synthase; 5573929 fatty acid synthase; 5575673 glutamyl aminopeptidase; 5575671 xanthine dehydrogenase;<br>5577897 argininosuccinate lyase; 5565274 argininosuccinate synthase; 23687459 alcohol dehydrogenase 2; 5577763 membrane-bound alkaline phosphatase; 5566353 D-3-phosphoglycerate dehydrogenase; 5574999 lopap isoform X2; 5578236 apolipoprotein D
aag02000 Transporters (11)	5578720 probable multidrug resistance-associated protein lethal(2)03659; 23687608 protein brown; 5567699 solute carrier family 2, facilitated glucose transporter member 3; 5577476 thiamine transporter 1; 110673989 mitochondrial basic amino acids transporter-like; 5570692 LOW QUALITY PROTEIN; 5572607 uncharacterized protein LOC5572607; 5564315LOW QUALITY PROTEIN; 5565788 facilitated trehalose transporter Tret1; 5579094 defensin-C; 5564141 MD-2-related lipid-recognition protein
aag01004 Lipid biosynthesis proteins (7)	5568814 fatty acid synthase; 5573929 fatty acid synthase; 5577524 acyl-CoA Delta(11) desaturase isoform X2; 5570274 elongation of very long chain fatty acids protein 4; 5575620 elongation of very long chain fatty acids protein 7; 5563869 elongation of very long chain fatty acids protein 7; 5564950 acyl-CoA synthetase family member 2, mitochondrial
aag04090 CD molecules (6)	5575673 glutamyl aminopeptidase; 5572429 CD109 antigen; 23687443 CD109 antigen; 5568826 CD109 antigen; 5572428 CD109 antigen; 5575000 perlucin-like protein
aag04030 G protein-coupled receptors (5)	5574685 gonadotropin-releasing hormone II receptor isoform X8, 5576882 opsin-3, 5568060 opsin-1; 5572198 opsin, ultraviolet-sensitive; 110680887 opsin-1-like
aag00199 Cytochrome P450 (4)	5569663 cytochrome P450 4d1 isoform X2; 5571193 probable cytochrome P450 6d5; 5571541 cytochrome P450 6d3; 5574850 cytochrome P450 302a1, mitochondrial
aag04131 Membrane trafficking (3)	5576429 low density lipoprotein receptor adapter protein 1-A; 5575000 perlucin-like protein; 5575673 glutamyl aminopeptidase
aag00537 Glycosylphosphatidylinositol (GPI)-anchored proteins (3)	5577763 membrane-bound alkaline phosphatase; 5574470 esterase B1; 5575056 chymotrypsin-like protease CTRL-1
aag01007 Amino acid related enzymes (3)	5572541 alanine aminotransferase 1; 5571325 tyrosine aminotransferase; 5576518 probable phosphoserine aminotransferase

aag04990 Domain-containing proteins not elsewhere classified (3)	5578083 spondin-1<br>5568942 nidogen-2<br>5575017 hemicentin-1
aag00536 Glycosaminoglycan binding proteins (2)	5565037 bombyxin B-1 homolog isoform X1<br>5563550 serine protease easter
aag04040 Ion channels (2)	5567215 pickpocket protein 28; 5566698 transient-receptor-potential-like protein
aag03036 Chromosome and associated proteins (2)	110677254 homeotic protein spalt-major isoform X1<br>5572250 group 3 secretory phospholipase A2 isoform X2
aag03000 Transcription factors (2)	5570966 LIM/homeobox protein Lhx9; 5566982 forkhead box protein E3 isoform X3
aag01001 Protein kinases (1)	5570893 dual specificity tyrosine-phosphorylation-regulated kinase 4 isoform X1
aag04052 Cytokines and growth factors (1)	5565037 bombyxin B-1 homolog isoform X1
aag03110 Chaperones and folding catalysts (1)	5564542 FK506-binding protein 2
aag04812 Cytoskeleton proteins (1)	5567446 gelsolin
aag04121 Ubiquitin system (1)	5566491 uncharacterized protein LOC5566491
aag01009 Protein phosphatases and associated proteins (1)	5570557 leucine-rich repeat-containing protein 23
aag04091 Lectins (1)	5575000 perlucin-like protein
aag03016 Transfer RNA biogenesis (1)	110677916 selenide, water dikinase 2-like isoform X1
aag04031 GTP-binding proteins (1)	5570133 guanine nucleotide-binding protein subunit beta-2
aag03037 Cilium and associated proteins (1)	5572577 rootletin isoform X1
aag04054 Pattern recognition receptors (1)	5575000 perlucin-like protein
