# Supplementary material for: Differences in gene expression in field populations of Wolbachia-infected Aedes aegypti mosquitoes with varying release histories in northern Australia
Source: PLoS Negl Trop Dis. 2023 Mar 29;17(3):e0011222. doi: 10.1371/journal.pntd.0011222 (PMC10085034; doi:10.1371/journal.pntd.0011222)
Supplement: S4 Table — (PDF) [file pntd.0011222.s005.pdf]

**S4 Table. Unmapped downregulated DEGs which were non-coding RNA from *Aedes aegypti* with different release years.**

| *Aedes aegypti* release year | | |
|---|---|---|
| **Aae.wMel$_{2011}$** | **Aae.wMel$_{2013/2014}$** | **Aae.wMel$_{2017}$** |
| LOC110674171, LOC110677214, LOC110674245, LOC110676610, LOC110674560, LOC110676459, LOC110678327, LOC110679860, LOC110678696, LOC110676194, LOC110679144, LOC110675307, LOC110677893, LOC110676333, LOC110674112 and LOC110674372 | LOC110679264, LOC110675798, LOC110679860, LOC110675419, LOC110678052, LOC110679845, LOC110678327, LOC110681560, LOC110677252, LOC110679144 and LOC110674112 | LOC110674426, LOC110675307, LOC110679848, LOC110677249, LOC110675921, LOC110679654, LOC110678978, LOC110679077, LOC110676416, LOC110675448, LOC110676459, LOC110680245, LOC110675798, LOC110674875, LOC110675739, LOC110676610, LOC110676947, LOC110674156, LOC110676068, LOC110674372, LOC110675480, LOC110680326, LOC110679406, LOC110678052, LOC110680549, LOC110678657, LOC110679051, LOC110678740, LOC110677977, LOC110675419, LOC110679845, LOC110676013, LOC110679264 and LOC110676333 |
