# Supplementary material for: Differences in gene expression in field populations of Wolbachia-infected Aedes aegypti mosquitoes with varying release histories in northern Australia
Source: PLoS Negl Trop Dis. 2023 Mar 29;17(3):e0011222. doi: 10.1371/journal.pntd.0011222 (PMC10085034; doi:10.1371/journal.pntd.0011222)
Supplement: S5 Table — (PDF) [file pntd.0011222.s006.pdf]

S5 Table. Unmapped downregulated DEGs in Aae.wMel$_{2011}$ mosquitoes which are not ncRNA.

Entrez Gene ID	Description/ Name	Function	Reference
110678281	pfam: Dimer_Tnp_hAT; hAT family C-terminal dimerisation region	Protein bares much similarity to zinc finger or transposase proteins	http://pfam.xfam.org/family/Dimer_Tnp_hAT
110676828	replication factor C subunit 3-like / Rfc5p, putative	DNA-directed DNA polymerase	https://www.ncbi.nlm.nih.gov/gene/?term=110676828
110674591	SMC_N; RecF/RecN/SMC N terminal domain	Structural maintenance of chromosomes_N Superfamily	https://www.ncbi.nlm.nih.gov/gene/?term=110674591
110680308	Protein Optix-like	May be involved in head or eye development; development of the clypeolabrum and several head sensory organs	https://www.uniprot.org/uniprot/Q95RW8
110675146	DNA Binding<br>Homologous to Deformed epidermal autoregulatory factor 1-like	When secreted, behaves as an inhibitor of cell proliferation, by arresting cells in the G0 or G1 phase.	https://www.uniprot.org/uniprot/A0A6I8U8T1<br>https://www.uniprot.org/uniprot/O75398
110676629	Uncharacterised		https://www.ncbi.nlm.nih.gov/gene/?term=110676629
110679076	Uncharacterised		https://www.ncbi.nlm.nih.gov/gene/?term=110679076
110680643	Carcinine transporter-like	CarT expression in photoreceptors is necessary and sufficient for fly vision and behavior.	https://www.ncbi.nlm.nih.gov/pmc/articles/PMC4739767/
110679455	Protein commissureless 2 homolog	Essential for nerve cord development. Functions downstream of fra to control axon guidance across the central nervous system (CNS) midline	https://www.ncbi.nlm.nih.gov/gene/?term=110679455,<br>https://www.uniprot.org/uniprot/Q9VUT8
CFI06_mgr02	16S ribosomal RNA	Translation process	https://www.ncbi.nlm.nih.gov/gene/?term=CFI06_mgr02
110679193	Putative oxidoreductase GLYR1 homolog	Oxidoreductase activity, positive regulation of histone acetylation, positive regulation of transcription by RNA polymerase II	https://www.ncbi.nlm.nih.gov/gene/?term=110679193,<br>https://www.uniprot.org/uniprot/Q8T079
110678839	Zinc finger C4H2 domain-containing protein-like	Plays a role in interneurons differentiation	https://www.ncbi.nlm.nih.gov/gene/?term=110678839,<br>https://www.uniprot.org/uniprot/Q9NQZ6
110675237	Apolipoprotein D-like	Apolipoprotein D (ApoD) is an extracellular glycoprotein of the lipocalin protein family, involved in different functions such as immune response, cell proliferation	https://www.ncbi.nlm.nih.gov/gene/?term=110675237,<br>https://www.intechopen.com/books/advances-in-lipoprotein-research/apolipoprotein-d

		regulation, chemoreception, retinoid metabolism, axon growth, and proteolysis regulation.	
110675476	Uncharacterised		https://www.ncbi.nlm.nih.gov/gene/?term=110675476
110680748	Zinc finger protein 569-like	ZNF569 protein may act as a transcriptional repressor that suppresses MAPK signaling pathway to mediate cellular functions.	https://www.ncbi.nlm.nih.gov/gene/?term=110680748, https://pubmed.ncbi.nlm.nih.gov/16793018/
110676559	BEN domain	BEN domain mediates protein-DNA and protein-protein interactions during chromatin organisation and transcription. The presence of BEN domains in a poxviral early virosomal protein and in polydnaviral proteins also suggests a possible role for them in organization of viral DNA during replication or transcription.	https://www.ncbi.nlm.nih.gov/gene/?term=110676559, https://www.ncbi.nlm.nih.gov/pmc/articles/PMC2477736/
110676930	Testis-specific zinc finger protein topi-like	The Drosophila aly-class meiotic arrest loci are essential for activation of transcription of many differentiation-specific genes, as well as several genes important for meiotic cell cycle progression, thus linking meiotic cell cycle progression to cellular differentiation during spermatogenesis.	https://www.ncbi.nlm.nih.gov/gene/?term=110676930, https://pubmed.ncbi.nlm.nih.gov/15084455/
110680210	Bromodomain adjacent to zinc finger domain protein 1A-like	Chromatin remodeling, DNA-dependent DNA replication, histone acetylation and regulation of transcription by RNA polymerase II	https://www.ncbi.nlm.nih.gov/gene/?term=110680210,
110679465	Uncharacterised		https://www.ncbi.nlm.nih.gov/gene/?term=110679465
110680634	PAB-dependent poly(A)-specific ribonuclease subunit PAN3-like	deadenylation-dependent decapping of nuclear-transcribed mRNA Source: GO_Central<br>mRNA processing, nuclear-transcribed mRNA poly(A) tail shortening, positive regulation of cytoplasmic mRNA processing body assembly	https://www.ncbi.nlm.nih.gov/gene/?term=110680634, https://www.uniprot.org/uniprot/B7Q0Q0
