# Supplementary material for: Differences in gene expression in field populations of Wolbachia-infected Aedes aegypti mosquitoes with varying release histories in northern Australia
Source: PLoS Negl Trop Dis. 2023 Mar 29;17(3):e0011222. doi: 10.1371/journal.pntd.0011222 (PMC10085034; doi:10.1371/journal.pntd.0011222)
Supplement: S6 Table — (PDF) [file pntd.0011222.s007.pdf]

S6 Table. Unmapped downregulated DEGs from Aae.wMel$_{2013/2014}$ mosquitoes.

Entrez Gene ID	Description	Function	Reference
LOC110678585	Paired box protein Pax-6-like	Involved in eye morphogenesis	https://www.ncbi.nlm.nih.gov/gene/?term=110678585, https://www.uniprot.org/uniprot/O18381
LOC110674017	Polyprenol reductase-like	N-Glycan biosynthesis	https://www.ncbi.nlm.nih.gov/gene/?term=110674017
LOC110676930	Testis-specific zinc finger protein topi-like	The Drosophila aly-class meiotic arrest loci are essential for activation of transcription of many differentiation-specific genes, as well as several genes important for meiotic cell cycle progression, thus linking meiotic cell cycle progression to cellular differentiation during spermatogenesis.	https://www.ncbi.nlm.nih.gov/gene/?term=110676930, https://pubmed.ncbi.nlm.nih.gov/15084455/
LOC110675182	Uncharacterised, BEN; BEN domain	?Organization of viral DNA during replication or transcription	https://www.ncbi.nlm.nih.gov/gene/?term=110675182, https://www.ncbi.nlm.nih.gov/pmc/articles/PMC2477736/
LOC110678581	Uncharacterized, MADF_DNA_bdg; Alcohol dehydrogenase transcription factor Myb/SANT-like	Transcription factor	https://www.ncbi.nlm.nih.gov/gene/?term=110678581, https://www.genome.jp/kegg-bin/get_htext
LOC110676965	Zinc finger BED domain-containing protein 1-like	Protein dimerization activity	https://www.ncbi.nlm.nih.gov/gene/?term=110676965, https://www.uniprot.org/uniprot/F6QDB5
LOC110674313	Zinc finger protein 845-like	Transcription factor	https://www.ncbi.nlm.nih.gov/gene/?term=110674313, https://www.genome.jp/kegg-bin/get_htext
LOC110678090	ATP-dependent DNA helicase PIF1-like	DNA replication proteins	https://www.ncbi.nlm.nih.gov/gene/?term=110678090, https://www.genome.jp/kegg-bin/get_htext?aag00001+5580192
CFI06_mgr02	16S ribosomal RNA	Translation process	https://www.ncbi.nlm.nih.gov/gene/?term=CFI06_mgr02
LOC110676559	BEN domain	BEN domain mediates protein-DNA and protein-protein interactions during chromatin organisation and transcription. The presence of BEN domains in a poxviral early virosomal protein and in polydnaviral proteins also suggests a possible role for them in organization of viral DNA during replication or transcription.	https://www.ncbi.nlm.nih.gov/gene/?term=110676559, https://www.ncbi.nlm.nih.gov/pmc/articles/PMC2477736/
LOC110675146	Deformed epidermal autoregulatory factor 1-like	Transcription factor	https://www.uniprot.org/uniprot/O75398
LOC110676076	Mantle protein-like	Protein Blast identified 98% similarity to Vajk2, which plays a role in chitin-based cuticle development in *Drosophila melanogaster*.	https://www.ncbi.nlm.nih.gov/gene/?term=110676076, https://www.uniprot.org/uniprot/Q8SZM2
