# Supplementary material for: Differences in gene expression in field populations of Wolbachia-infected Aedes aegypti mosquitoes with varying release histories in northern Australia
Source: PLoS Negl Trop Dis. 2023 Mar 29;17(3):e0011222. doi: 10.1371/journal.pntd.0011222 (PMC10085034; doi:10.1371/journal.pntd.0011222)
Supplement: S7 Table — (PDF) [file pntd.0011222.s008.pdf]

S7 Table. Unmapped downregulated DEGs in Aae.wMel$_{2017}$ mosquitoes.

Gene ID	Description	Function	Reference
LOC110675237	40S ribosomal protein S17	Translation	https://www.ncbi.nlm.nih.gov/gene/?term=110680939, https://www.uniprot.org/uniprot/Q52UT2
LOC110678090	apolipoprotein D-like		https://www.ncbi.nlm.nih.gov/gene/?term=110675237
LOC110675182	ATP-dependent DNA helicase PIF1-like	DNA-dependent ATPase and 5'-3' DNA helicase required for the maintenance of both mitochondrial and nuclear genome stability	https://www.ncbi.nlm.nih.gov/gene/?term=110678090, https://www.uniprot.org/uniprot/Q9H611
LOC110677148	BEN domain		https://www.ncbi.nlm.nih.gov/gene/?term=110675182%5Buid%5D
LOC110680907	cell division cycle protein 20 homolog	This protein is involved in the pathway protein ubiquitination, which is part of Protein modification.	https://www.ncbi.nlm.nih.gov/gene/?term=110677148, https://www.uniprot.org/uniprot/Q12834
LOC110674064	CREB-regulated transcription coactivator 1-like	cAMP-responsive element binding protein (CREB) has been well known as one of the best studied inducible eukaryotic transcription factors.	https://www.ncbi.nlm.nih.gov/gene/?term=110680907, https://pubmed.ncbi.nlm.nih.gov/25896045/
LOC110680411	DNA repair protein XRCC3-like	Involved in the homologous recombination repair (HRR) pathway of double-stranded DNA, thought to repair chromosomal fragmentation, translocations, and deletions.	https://www.ncbi.nlm.nih.gov/gene/?term=110674064, https://www.uniprot.org/uniprot/O43542
LOC110674134	Domain of unknown function (DUF4806)		https://www.ncbi.nlm.nih.gov/gene/?term=110680411
LOC110680252	dynein heavy chain 10, axonemal	Force generating protein of respiratory cilia. Produces force towards the minus ends of microtubules. Dynein has ATPase activity	https://www.ncbi.nlm.nih.gov/gene/?term=110674134, https://www.uniprot.org/uniprot/Q8IVF4
LOC110674232	E3 SUMO-protein ligase PIAS2-like	Mosquito SUMOylation pathway plays a broadly antiviral role against a wide range of clinically important arboviruses, including Zika, Semliki Forest, and Bunyamwera viruses. SUMO important in post-translational modification.	https://www.ncbi.nlm.nih.gov/gene/?term=110680252, https://journals.plos.org/plospathogens/article?id=10.1371/journal.ppat.1009134,
LOC110676122	Ephexin Pleckstrin homology (PH) domain	PH domains play a role in recruiting proteins to different membranes, thus targeting them to appropriate cellular compartments or enabling them to interact with other components of the signal transduction pathways.	https://www.ncbi.nlm.nih.gov/gene/?term=110674232, https://www.ebi.ac.uk/interpro/entry/InterPro/IPR001849/
LOC110673995	fer-1-like protein 6	transcription factors as being involved in *Odorant receptor* regulation	https://www.ncbi.nlm.nih.gov/gene/?term=110676122, https://bmcgenomics.biomedcentral.com/articles/10.1186/s12864-020-07336-w

LOC110679476	FLYWCH zinc finger domain	This domain was first characterised in Drosophila Modifier of mdg4 proteins, Mod(mgd4), putative chromatin modulators involved in higher order chromatin domains.	https://www.ebi.ac.uk/interpro/entry/InterPro/IPR007588/
LOC110678281	hAT family C-terminal dimerisation region	the protein bares much similarity to zinc finger or transposase proteins.	https://www.ncbi.nlm.nih.gov/gene/?term=110679476, http://pfam.xfam.org/family/Dimer_Tnp_hAT
LOC110674011	hAT family C-terminal dimerisation region		https://www.ncbi.nlm.nih.gov/gene/?term=110678281
LOC110675487	Headcase protein-like	Required for imaginal cell differentiation, may be involved in hormonal responsiveness during metamorphosis (34), headcase was identified in a screen as a regulator of the siRNA pathway (35)	https://www.ncbi.nlm.nih.gov/gene/?term=110674011
LOC110676735	histone deacetylase Rpd3-like	Histone deacetylation plays an important role in transcriptional regulation, cell cycle progression, DNA damage response, osmotic stress response and developmental events,	https://www.ncbi.nlm.nih.gov/gene/?term=110675487, https://www.uniprot.org/uniprot/P32561
LOC110675977	histone H4	Sense and antisense histone 4-derived piRNAs accumulate in an Ago3/Piwi5 ping-pong-dependent fashion	https://www.ncbi.nlm.nih.gov/gene/?term=110676735, https://www.jimmunol.org/content/jimmunol/190/2/650.full.pdf?with-ds=yes
LOC110680300	lactosylceramide 1,3-N-acetyl-beta-D-glucosaminyltransferase	sphingolipid biosynthesis, plays a key role in the synthesis of lacto- or neolacto-series carbohydrate chains on glycolipids	https://www.ncbi.nlm.nih.gov/gene/?term=110675977, https://journals.plos.org/plosone/article/file?type=supplementary&id=info:doi/10.1371/journal.pone.0155616.s005 Massive Shift in Gene Expression during Transitions between Developmental Stages of the Gall Midge, Mayetiola Destructor
LOC110678193	leucine-rich repeat [structural motif]		https://www.ncbi.nlm.nih.gov/gene/?term=110680300
LOC110679126	lipid storage droplets surface-binding protein 2-like	Several RNA viruses use host LDs at different steps of their life cycle	https://www.ncbi.nlm.nih.gov/gene/?term=110678193, https://www.ncbi.nlm.nih.gov/pmc/articles/PMC3268388/
LOC110678581	LRR_AMN1; leucine-rich repeat [structural motif]	Immune	https://www.ncbi.nlm.nih.gov/gene/?term=110679126
LOC110677913	MADF_DNA_bdg; Alcohol dehydrogenase	regulation of transcription, DNA-templated	https://www.ncbi.nlm.nih.gov/gene/?term=110678581, https://www.uniprot.org/uniprot/Q9LV59

	transcription factor Myb/SANT-like		
LOC110679883	Major facilitator superfamily domain-containing protein 10	apoptotic process, sodium-independent organic anion transport	https://www.ncbi.nlm.nih.gov/gene/?term=110677913, https://www.uniprot.org/uniprot/Q14728
LOC110679883	Mucin-5AC-like	Amino sugar and nucleotide sugar metabolism	https://www.ncbi.nlm.nih.gov/gene/?term=110679883, https://www.genome.jp/kegg-bin/get_htext
LOC110680249	oocyte zinc finger protein XlCOF22-like	Transcription, Transcription regulation	https://www.ncbi.nlm.nih.gov/gene/?term=110680249
LOC110680634	PAB-dependent poly(A)-specific ribonuclease subunit PAN3-like	mRNA surveillance and transport factors	https://www.ncbi.nlm.nih.gov/gene/?term=110680634, https://www.genome.jp/kegg-bin/get_htext?aag03019+5577183
LOC110678585	Paired box protein Pax-6-like	Involved in eye morphogenesis and adult development	https://www.ncbi.nlm.nih.gov/gene/?term=110678585, https://www.uniprot.org/uniprot/O18381
LOC110676083	Probable E3 ubiquitin protein ligase DRIPH	This protein is involved in the pathway protein ubiquitination, which is part of Protein modification.	https://www.ncbi.nlm.nih.gov/gene/?term=110676083, https://www.uniprot.org/uniprot/Q9LS86
LOC110680850	Proline-rich protein 36	This gene encodes a large protein of unknown function that contains internal regions of low complexity sequence. Alternative splicing results in multiple transcript variants. The transcript structure of the protein-coding variant at this locus is conserved between human and mouse	https://www.ncbi.nlm.nih.gov/gene/?term=110680850, https://www.genecards.org/cgi-bin/carddisp.pl?gene=PRR36
LOC110679455	Protein commissureless 2 homolog	Essential for nerve cord development. Functions downstream of fra to control axon guidance across the central nervous system (CNS) midline	https://www.ncbi.nlm.nih.gov/gene/?term=110679455, https://www.uniprot.org/uniprot/Q9VUT8
LOC110676568	Putative defense protein Hdd11-like	May have antimicrobial activity, defense response to bacterium, defense response to protozoan, innate immune response	https://www.ncbi.nlm.nih.gov/gene/?term=110676568, https://www.uniprot.org/uniprot/Q86RS3
LOC110680234	RecF/RecN/SMC N terminal domain	function together with other proteins in a range of chromosomal transactions, including chromosome condensation, sister-chromatid cohesion, recombination, DNA repair and epigenetic silencing of gene expression	https://www.ncbi.nlm.nih.gov/gene/?term=110680234, https://www.ebi.ac.uk/interpro/entry/InterPro/IPR003395/
LOC110676828	replication factor C subunit 3-like	May be involved in DNA replication and thus regulate cell proliferation	https://www.ncbi.nlm.nih.gov/gene/?term=110676828, https://www.uniprot.org/uniprot/Q8VXX4
LOC110674773	Sec63; Sec63 Brl domain	Proteins destined for the secretory pathway are initially translocated across the membrane of the endoplasmic	https://www.ncbi.nlm.nih.gov/gene/?term=110674773

		reticulum (ER)4 at pore-forming structures known as translocons. A substantial body of data indicates that Sec63p and Kar2p contribute directly to the driving force for post-translational translocation, with the ATPase activity of Kar2p being activated by the luminal J-domain within Sec63p (9, 10). basic function in translocation	
LOC110678733	serine proteinase stubble	Trypsin	https://www.ncbi.nlm.nih.gov/gene/?term=110678733, https://www.genome.jp/dbget-bin/www_bget?aag:5572015
LOC110680822	set1/Ash2 histone methyltransferase complex subunit ASH2-like	Transcriptional regulator	https://www.ncbi.nlm.nih.gov/gene/?term=110680822, https://www.uniprot.org/uniprot/Q9UBL3
LOC110677032	short stature homeobox protein 2-like	May be a growth regulator and have a role in specifying neural systems involved in processing somatosensory information, as well as in face and body structure formation.	https://www.ncbi.nlm.nih.gov/gene/?term=110677032, https://www.uniprot.org/uniprot/O60902
LOC110679408	Tc5 transposase DNA-binding domain	DNA-binding	https://www.ncbi.nlm.nih.gov/gene/?term=110679408, https://www.uniprot.org/uniprot/Q23E04
LOC110675561	tektin-1	Tektins are insoluble α-helical proteins essential for the construction of cilia and flagella and are found throughout the eukaryotes apart from higher plants.	https://www.ncbi.nlm.nih.gov/gene/?term=110675561, https://genomebiology.biomedcentral.com/articles/10.1186/gb-2008-9-7-229
LOC110676106	tubulin alpha-8 chain-like	Tubulin is the major constituent of microtubules.	https://www.ncbi.nlm.nih.gov/gene/?term=110676106, https://www.uniprot.org/uniprot/Q9NY65
LOC110679470	Tudor domain	TUDOR-domain containing (Tudor) proteins facilitate piRNA biogenesis in Drosophila melanogaster and other model organisms.	https://www.ncbi.nlm.nih.gov/gene/?term=110679470, https://www.researchgate.net/publication/329844811_The_Tudor_protein_Veneno_assembles_the_ping-pong_amplification_complex_that_produces_viral_piRNAs_in_Aedes_mosquitoes
LOC110681183	WASH complex subunit 2-like	protein localization to endosome, protein transport, retrograde transport, endosome to Golgi	https://www.ncbi.nlm.nih.gov/gene/?term=110681183, https://www.uniprot.org/uniprot/Q6PGL7
LOC110674056	Werner syndrome ATP-dependent helicase-like	May play an important role in the dissociation of joint DNA molecules that can arise as products of homologous recombination, at stalled replication forks or during DNA repair. Alleviates stalling of DNA polymerases at the site of DNA lesions. Important for genomic integrity. Plays a role in the formation of DNA replication focal centers;	https://www.ncbi.nlm.nih.gov/gene/?term=110674056, https://www.uniprot.org/uniprot/Q14191

		stably associates with foci elements generating binding sites for RP-A (By similarity). Plays a role in double-strand break repair after gamma-irradiation.	
LOC110680251	XPG domain containing	DNA repair	https://www.ncbi.nlm.nih.gov/gene/?term=110680251, https://www.uniprot.org/uniprot/A0A1S4EVT1
LOC110676965	zinc finger BED domain-containing protein 1-like	DNA binding	https://www.ncbi.nlm.nih.gov/gene/?term=110676965, https://www.uniprot.org/uniprot/F6QDB5
LOC110681552	zinc finger protein 148-like	Involved in transcriptional regulation. Represses the transcription of a number of genes including gastrin, stromelysin and enolase. Binds to the G-rich box in the enhancer region of these genes.	https://www.ncbi.nlm.nih.gov/gene/?term=110681552, https://www.uniprot.org/uniprot/Q9UQR1
LOC110680748	zinc finger protein 569-like	May be involved in transcriptional regulation	https://www.ncbi.nlm.nih.gov/gene/?term=110680748, https://www.uniprot.org/uniprot/Q5MCW4#function
LOC110674313	zinc finger protein 845-like	May be involved in transcriptional regulation.	https://www.ncbi.nlm.nih.gov/gene/?term=110674313, https://www.genecards.org/cgi-bin/carddisp.pl?gene=ZNF845
LOC110679831	zinc finger protein DZIP1L	Involved in primary cilium formation	https://www.ncbi.nlm.nih.gov/gene/?term=110679831, https://www.uniprot.org/uniprot/Q8IYY4
