# Supplementary material for: Differences in gene expression in field populations of Wolbachia-infected Aedes aegypti mosquitoes with varying release histories in northern Australia
Source: PLoS Negl Trop Dis. 2023 Mar 29;17(3):e0011222. doi: 10.1371/journal.pntd.0011222 (PMC10085034; doi:10.1371/journal.pntd.0011222)
Supplement: S1 Box — (PDF) [file pntd.0011222.s009.pdf]

S1 Box. Significantly enriched GO terms pertaining to biological processes and molecular functions in Aae.wMel$_{2017}$ mosquitoes and KEGG pathways in Aae.wMel$_{2013-14}$ and Aae.wMel$_{2017}$ mosquitoes.

A) Significantly enriched biological processes in Aae.wMel$_{2017}$ mosquitoes

GO:0006270 DNA replication initiation
GO:0006260 DNA replication
GO:0060070 canonical Wnt signaling pathway
GO:0000724 double-strand break repair via homologous recombination
GO:0006281 DNA repair
GO:0006979 response to oxidative stress
GO:0006334 nucleosome assembly
GO:0007275 multicellular organism development
GO:0042438 melanin biosynthetic process
GO:0060179 male mating behavior
GO:0071139 resolution of recombination intermediates
GO:0007018 microtubule-based movement

B) Significantly enriched molecular functions in Aae.wMel$_{2017}$ mosquitoes

GO:0005524 ATP binding
GO:0004672 protein kinase activity
GO:0003684 damaged DNA binding
GO:0004601 peroxidase activity
GO:0003678 DNA helicase activity
GO:0042813 Wnt-activated receptor activity
GO:0004674 protein serine/threonine kinase activity
GO:0008270 zinc ion binding
GO:0003777 microtubule motor activity

C) KEGG pathways identified by KEGG mapper in Aae.wMel$_{2013/2014}$ mosquitoes*

aag04080 Neuroactive ligand-receptor interaction (3)
aag01100 Metabolic pathways (2)
aag04213 Longevity regulating pathway - multiple species (1)
aag00510 N-Glycan biosynthesis (1)
aag04320 Dorso-ventral axis formation (1)
aag04150 mTOR signaling pathway (1)
aag00280 Valine, leucine and isoleucine degradation (1)
aag04310 Wnt signaling pathway (1)
aag00532 Glycosaminoglycan biosynthesis - chondroitin sulfate / dermatan sulfate (1)
aag00310 Lysine degradation (1)
aag04330 Notch signaling pathway (1)
aag04141 Protein processing in endoplasmic reticulum (1)

* KEGG pathway ID, Pathway name and number of genes identified from our DEG List in brackets.

D) KEGG pathways identified by KEGG mapper in Aae.wMel$_{2017}$ mosquitoes*

aag01100 Metabolic pathways (12)
aag04310 Wnt signaling pathway (10)
aag03030 DNA replication (6)
aag03460 Fanconi anemia pathway (6)
aag04068 FoxO signaling pathway (5)
aag03420 Nucleotide excision repair (4)
aag04013 MAPK signaling pathway (4)
aag04150 mTOR signaling pathway (4)
aag04080 Neuroactive ligand-receptor interaction (4)
aag03430 Mismatch repair (4)
aag01240 Biosynthesis of cofactors (3)
aag04120 Ubiquitin mediated proteolysis (3)
aag04330 Notch signaling pathway (3)
aag04320 Dorso-ventral axis formation (3)
aag04145 Phagosome (2)
aag00981 Insect hormone biosynthesis (2)
aag04341 Hedgehog signaling pathway - fly (2)
aag03440 Homologous recombination (2)
aag03410 Base excision repair (2)
aag00790 Folate biosynthesis (2)
aag04213 Longevity regulating pathway (2)
aag00983 Drug metabolism - other enzymes (2)
aag03022 Basal transcription factors 2)
aag03010 Ribosome (1)
aag00982 Drug metabolism - cytochrome P450 (1)
aag00280 Valine, leucine and isoleucine degradation (1)
aag00860 Porphyrin and chlorophyll metabolism (1)
aag04624 Toll and Imd signaling pathway (1)
aag00010 Glycolysis / Gluconeogenesis (1)
aag00730 Thiamine metabolism (1)
aag00830 Retinol metabolism (1)
aag00053 Ascorbate and aldarate metabolism (1)
aag03018 RNA degradation (1)
aag04140 Autophagy - animal (1)
aag00562 Inositol phosphate metabolism (1)
aag00020 Citrate cycle (TCA cycle) (1)
aag00240 Pyrimidine metabolism (1)
aag00480 Glutathione metabolism (1)
aag04141 Protein processing in endoplasmic reticulum (1)
aag00500 Starch and sucrose metabolism (1)
aag00040 Pentose and glucuronate interconversions (1)
aag00330 Arginine and proline metabolism (1)
aag00620 Pyruvate metabolism (1)
aag04215 Apoptosis - multiple species (1)
aag00600 Sphingolipid metabolism (1)
aag00310 Lysine degradation (1)
aag04392 Hippo signaling pathway - multiple species (1)
aag00130 Ubiquinone and other terpenoid-quinone biosynthesis (1)
aag04144 Endocytosis (1)
aag04070 Phosphatidylinositol signaling system (1)
aag00230 Purine metabolism (1)
aag03040 Spliceosome (1)
aag04214 Apoptosis - fly (1)
aag03015 mRNA surveillance pathway (1)
aag00350 Tyrosine metabolism (1)
aag04137 Mitophagy - animal (1)
aag04391 Hippo signaling pathway - fly (1)
aag00980 Metabolism of xenobiotics by cytochrome P450 (1)
